# Effects of nonsocial and circumscribed interest images on neural mechanisms of emotion regulation in autistic adults

**DOI:** 10.3389/fnbeh.2022.1057736

**Published:** 2022-12-07

**Authors:** Ligia Antezana, Marika C. Coffman, Antoinette Sabatino DiCriscio, John A. Richey

**Affiliations:** ^1^Department of Psychology, Virginia Tech, Blacksburg, VA, United States; ^2^Department of Psychiatry, University of Pittsburgh, Pittsburgh, PA, United States; ^3^Duke Center for Autism and Brain Development, Duke University, Durham, NC, United States; ^4^Department of Psychiatry and Behavioral Sciences, Duke University, Durham, NC, United States; ^5^Geisinger Health System, Geisinger Autism & Developmental Medicine Institute (ADMI), Lewisburg, PA, United States

**Keywords:** autism, nonsocial, circumscribed interests, emotion regulation, cognitive reappraisal

## Abstract

**Introduction:**

Emotion dysregulation is commonly reported among autistic individuals. Prior work investigating the neurofunctional mechanisms of emotion regulation (ER) in autistic adults has illustrated alterations in dorsolateral prefrontal cortex (dlPFC) activity, as well as concurrent atypical patterns of activation in subcortical regions related to affect during cognitive reappraisal of social images. Whereas most research examining ER in autism has focused on regulation of negative emotions, the effects of regulating positive emotions has been generally understudied. This is surprising given the relevance of positive motivational states to understanding circumscribed interests (CI) in autism.

**Methods:**

Accordingly, the purpose of this study was to use fMRI with simultaneous eye-tracking and pupillometry to investigate the neural mechanisms of ER during passive viewing and cognitive reappraisal of a standardized set of nonsocial images and personalized (self-selected) CI images.

**Results:**

The autistic group demonstrated comparatively reduced modulation of posterior cingulate cortex (PCC) activation during cognitive reappraisal of CI images compared to viewing of CI, although no eye-tracking/pupillometry differences emerged between-groups. Further, the autistic group demonstrated increased PCC connectivity with left lateral occipital and right supramarginal areas when engaging in cognitive reappraisal vs. viewing CI.

**Discussion:**

In autistic adults, CI may be differentially modulated *via* PCC. Considering the documented role of the PCC as a core hub of the default mode network, we further postulate that ER of CI could potentially be related to self-referential cognition.

## Introduction

Emotion regulation (ER) processes involve modulation of cognitive, behavioral, and physiological aspects of emotion (Bargh and Williams, 2007; Rottenberg and Gross, 2007), and are linked to specific cognitive strategies such as reappraisal, acceptance, rumination, and avoidance (Aldao et al., 2010). These cognitive strategies influence both the valence and intensity of emotion (Ochsner et al., 2002), which have in turn been associated with increasingly well-defined patterns of brain activation, mainly (although not exclusively) involving the dorsolateral prefrontal cortex (dlPFC; Ochsner et al., 2002; Ochsner and Gross, 2005; McRae et al., 2012). According to DSM-5 criteria, autism spectrum disorder is characterized by two broad domains, which include difficulties in social communication and interaction and restricted and repetitive behaviors and interests (RRBI; American Psychiatric Association, 2013). Although not a core diagnostic feature of autism, difficulties in regulating emotions are frequently reported clinically (Lecavalier, 2006; Totsika et al., 2011), and have been linked to development of co-occurring internalizing and externalizing difficulties (Mazefsky and White, 2014; Rieffe et al., 2014; White et al., 2014). In light of its potential explanatory role in certain features of autism (i.e., physiological arousal, affective differences, rigidity), emotion dysregulation has been introduced as a general framework for understanding observations of emotional reactivity and heightened emotional responses to certain stimuli (Mazefsky et al., 2013; White et al., 2014). Neuroimaging work has elucidated altered patterns of prefrontal cortex activation in autistic individuals when regulating emotional responses (Pitskel et al., 2014; Richey et al., 2015). To date, most prior research examining the nature of ER mechanisms in autism has focused mainly on regulation of negative (i.e., disgust stimuli) and social stimuli (Pitskel et al., 2014; Richey et al., 2015), no work has characterized the effects of regulating highly positive stimuli, which is surprising given the relevance of this process to core diagnostic features of autism such as high motivation toward topics of intense fascination or “circumscribed interests” (CI). Accordingly, the purpose of this study was to use fMRI during simultaneous eye-tracking/pupillometry assessment to characterize the neurofunctional mechanisms of ER during cognitive reappraisal and passive viewing of personalized CI images.

Cognitive reappraisal is one of several ER strategies involving changes in the way one interprets a stimulus in order to modify its affective impact (Gross, 1998). Prior research has identified cognitive reappraisal as a particularly adaptive ER strategy, insofar as it has been specifically related to better psychological health (Hopp et al., 2011), overall well-being (Haga et al., 2009), and negatively linked to psychopathology (Aldao and Nolen-Hoeksema, 2010). Conversely, passive ER strategies (e.g., avoidance, rumination) have been linked with increased psychopathology (Aldao and Dixon-Gordon, 2014), and increased correlates of stress (Lewis et al., 2018). When examining the landscape of ER strategies in autistic samples, research in primarily child and adolescent samples has found that compared to neurotypical peers, autistic individuals reported and demonstrated less use of adaptive ER strategies, i.e., cognitive reappraisal and problem-solving strategies (Samson et al., 2012, 2015a,b,c). Further, autistic individuals have been found to frequently rely on maladaptive ER strategies, i.e., avoidance, emotion suppression, and rumination as compared to non-autistic peers (Jahromi et al., 2012; Mazefsky et al., 2014; Patel et al., 2017; Charlton et al., 2019). Thus, cognitive reappraisal appears to be an ER strategy that is particularly adaptive and also generally underutilized by autistic people. Additionally, it has been posited that core autistic traits may uniquely interact with ER (Mazefsky et al., 2012; Nuske et al., 2013). Previous work has frequently found associations between ER difficulties and autistic traits (Samson et al., 2014; Berkovits et al., 2017), with the strongest associations to RRBI (Samson et al., 2014; Greenlee et al., 2021; Martínez-González et al., 2021).

Specific RRBI facets such as hand-flapping, body rocking, ritualistic and self-injurious behaviors are theorized as emotion regulation strategies (Gabriels et al., 2013; Samson et al., 2015c; Kapp et al., 2019). Within the RRBI domain a particularly understudied core feature that may have specific linkage to ER is circumscribed interests (CI), defined as intense preoccupations or fascinations of a specific focus or topic, which can be interfering in nature (Turner-Brown et al., 2011). Recent work in autistic adults has also highlighted that engaging in CI may be an emotion regulation strategy that can prevent negative outcomes (Grove et al., 2018; Raymaker et al., 2020; Higgins et al., 2021; Mantzalas et al., 2022a,b). Thus, links between increased engagement in CI and greater internalizing difficulties may be in part related to increased use of CI as an emotion regulation strategy (Grove et al., 2018). Previous work examining CI has used personalized CI images, as well as nonsocial object images (Sasson et al., 2008), which are preferred by autistic people over social images and considered a proxy for CI. This work has demonstrated that CI are highly salient and rewarding for autistic individuals (Sasson et al., 2008; Dichter et al., 2012; Kohls et al., 2013, 2018). Additionally, the hyper-motivational significance of CI may disproportionately engage and deplete finite cognitive resources making it difficult to flexibly shift and adaptively respond to other information (Mazefsky et al., 2012; White et al., 2014; Richey et al., 2015). CI further appear to comprise certain characteristics that make them distinct from preferred interests in neurotypical peers; for example parents of autistic youth rated their children’s interests with higher intensity vs. typically-developing controls (i.e., more interfering, time-consuming, higher degree of resistance when interrupted; Anthony et al., 2013). Further, Sasson et al. (2012) found that autistic adults reported higher affective ratings toward common nonsocial object images than neurotypical adults, suggesting a systematic difference in motivational patterns based on the social vs. nonsocial nature of environmental stimuli. Similarly, autistic adults have reported higher intrinsic motivation toward their interests compared to neurotypical adults (Grove et al., 2016). These self- and parent-reported measures of interests in autistic people converge with atypical behavioral patterns noted in the context of CI information (i.e., increased attention, saliency of CI). Despite the intensity of CI, and its potential to impact ER, neural patterns of ER with regard to preferred interests have yet to be examined.

Neural mechanisms of motivation and ER are tightly intertwined, sharing common activation and co-activation patterns (Brandl et al., 2019). Discrepant hypo- and hyper-motivational tendencies toward social and CI information in autism, as posited by the motivation hypothesis (Chevallier et al., 2012; Dichter et al., 2012; Clements et al., 2018), may be related to ER abilities, such that affect toward such information may be difficult to regulate through prefrontally situated systems. This notion is consistent with work that has established that motivation toward social and nonsocial incentives in autistic people moderated the impact of autistic traits on emotional health outcomes (Han et al., 2019). Thus, the motivational tendencies and substrates that underlie autistic traits (c.f., the motivation hypothesis; Clements et al., 2018, 2022; Dichter, 2018) may add to the neurobiological vulnerabilities that potentiate ER differences for autism (White et al., 2014; Richey et al., 2015; Cai et al., 2018).

There is a growing literature examining the role of affect, reward, and attention toward nonsocial object information (i.e., trains, computers) in autism using physiological, eye tracking, and behavioral performance patterns with recent work extending this to personalized CI. Studies using eye tracking have found that autistic individuals preferentially attend to nonsocial objects vs. social images compared to neurotypical individuals (Sasson et al., 2008, 2011). Additionally, one study found that autistic children tended to perform similarly to matched typically-developing children in visual attention to faces in the context of object images, but when faces were in the context of CI-related images, they attended to faces significantly less than typically-developing children (Sasson and Touchstone, 2014). Moreover, autistic adults demonstrate greater arousal, as evidenced by greater pupil size and blink rate, toward nonsocial object images compared to neurotypical adults (Traynor et al., 2019). Recent work by Bos et al. (2019) found that personalized interest cues altered behavioral inhibition within a go/no-go task for autistic children vs. typically-developing children, such that autistic children made more mistakes when personalized interests were used as cues. Altogether, there is evidence to suggest that nonsocial object and CI information may alter attentional and arousal mechanisms for autism, further suggesting that affect toward hyper-motivational information may impact ER mechanisms in autistic people.

Heightened arousal and attention patterns toward nonsocial object and CI information in autism have been linked to neural alterations of brain regions associated with salience and reward (Langen et al., 2011a,b; Yerys, 2015). Specifically, two studies have found increased activation in regions associated with reward during incentive delay tasks with interest cues in autistic individuals compared to neurotypical individuals (Dichter et al., 2012; Kohls et al., 2013). Moreover, compared to typically-developing children, autistic children demonstrated greater activation of regions associated with the salience network (Cascio et al., 2014) and areas of visual expertise (Foss-Feig et al., 2016) when viewing personalized interest images vs. other’s interest images. Further, emerging work with resting state functional connectivity in networks associated with cognitive control, reward, and salience have been found to index RRBI. For example, increased RRBI was associated with increased connectivity of the salience network and imbalance between limbic vs. cognitive and motor circuitry (Uddin et al., 2013; Abbott et al., 2018). Thus, the prefrontal-dependent inhibitory systems that relate to altered attention, affect, and reward toward CI may impact ER mechanisms.

Despite growing literature implicating altered prefrontal mechanisms with ER in autism (Mazefsky et al., 2013, 2020; White et al., 2014; Richey et al., 2015; Cai et al., 2018), only two fMRI studies have examined neural mechanisms of cognitive reappraisal in autistic people. The cognitive reappraisal task has been used to examine ER, in this task participants are instructed to upregulate (“Think Positive”) or downregulate (“Think Negative”) their affect toward neutral or emotionally-valenced stimuli (Pitskel et al., 2014; Richey et al., 2015). These studies found that compared to neurotypical individuals, autistic individuals demonstrated decreased ability in regulating limbic (i.e., amygdala, insula) reactivity *via* prefrontally-situated systems when engaging in cognitive reappraisal conditions vs. natural viewing toward social (Richey et al., 2015) and disgust (Pitskel et al., 2014) images. Although ER mechanisms have been examined for generally hypo-motivational stimuli, it is not known as to whether these same ER mechanisms are implicated in regulating hyper-motivational stimuli (i.e., CI and nonsocial object images) in autism, and further if personalized CI uniquely effect ER mechanisms as compared to nonsocial object information.

In order to investigate the role of ER in hyper-motivational contexts in autism, this study examined mechanisms of cognitive reappraisal in the context of personalized CI and nonsocial object images. To examine ER, participants completed a cognitive reappraisal task while viewing personalized CI images compared to nonsocial objects (i.e., trains, computers) during simultaneous eye-tracking, pupillometry and fMRI data collection. We hypothesized the following: (1) consistent with cognitive reappraisal literature, the autistic group would show diminished dorsolateral prefrontal cortex (dlPFC) activation during ER of both nonsocial and CI stimuli compared to the neurotypical group, (2) consistent with reward literature, the autistic group would demonstrate greater activation in striatal areas before regulating their thoughts during passive viewing of their personalized CI compared to when viewing nonsocial object stimuli, (3) behavioral reports of RRBI would be related to neural activation of striatal regions when viewing and regulating thoughts about personalized CI in the autistic group, and (4) behavioral responses (i.e., self-report, eye-tracking and pupillary metrics) would show greater motivation and preference toward CI objects compared to nonsocial objects in the autistic group vs. neurotypical group.

## Materials and methods

### Participants and procedures

Participants consented to protocols approved by the local Human Investigations Committees at both UNC-Chapel Hill and Duke University Medical Centers. Sixteen right-handed autistic adult participants were recruited from the Autism Subject Registry through the UNC Carolina Institute for Developmental Disabilities; a matched group of 16 neurotypical adult participants were recruited *via* the Duke-UNC Brain Imaging and Analysis Center (BIAC) subject lists (Table 1). For every autistic participant, a neurotypical participant was matched on a one-to-one basis along several factors (i.e., sex assigned at birth, race, ethnicity, age within ± one year, and IQ within ± five points). Neurotypical participants were not taking any psychotropic medications at the time of scanning. All participants had normal or corrected-to-normal vision, and exclusion criteria for the autistic group included a history of medical conditions, including Fragile X syndrome, tuberous sclerosis, neurofibromatosis, phenylketonuria, epilepsy, and gross brain injury, IQ < 80, or MRI contraindications. One autistic participant was dropped from analysis due to report of not following task instructions, thus the final sample presented here is representative of the 15 autistic participants and 16 neurotypical participants (Table 1). Regarding race and ethnicity, the neurotypical sample consisted of two participants who self-identified as Asian, one participant who self-identified as Black, and 13 participants that self-identified as White. In the autistic group, two participants self-identified as Asian, one participant self-identified as Black and 12 participants self-identified as White. No participants reported Hispanic or Latino ethnicity. Additionally, the majority of the sample was right-handed, and one participant per group was left-handed.

**TABLE 1:** Participant characteristics.

	Autistic group *n* = 15 *M* (SD)	Neurotypical group *n* = 16 *M* (SD)	*P*-value
Age (years)	26.14 (8.13)	25.82 (7.00)	0.91
Range	18.25–43.92	19.08–42.92	
FSIQ-4 (SS)	113.60 (13.77)	116.25 (12.12)	0.57
Range	79–128	95–134	
Sex Assigned at Birth (M:F)	13:2	14:2	0.94
ADOS-G Total Score	15.4 (6.94)	–	–
Range	8–37		
ADOS-G Communication	5.33 (4.29)	–	–
Range	2–20		
ADOS-G Social Interaction	8.07 (2.37)	–	–
Range	4–12		
ADOS-G Stereotyped Behaviors and Restricted Interests	2.00 (1.69)	–	–
Range	0–5	–	–
IRB Total Score	21.60 (6.28)	3.69 (4.19)	<0.001
Range	13–32	0–13	
IRB Motor Stereotypies Score	6.80 (3.61)	0.88 (1.41)	<0.001
Range	0–13	0–4	
IRB Insistence on Sameness Score	6.33 (3.68)	0.38 (0.89)	<0.001
Range	0–14	0–3	
IRB Circumscribed Interests Score	8.47 (6.27)	2.43 (2.94)	<0.001
Range	4–13	0–9	
RBS-R Total Score	38.07 (22.84)	9.38 (14.03)	<0.001
Range	11–75	0–50	
RBS-R Stereotyped Score	5.44 (3.54)	2.13 (3.32)	0.01
Range	1–11	0–8	
RBS-R Self-Injurious Score	2.67 (2.30)	0.44 (1.27)	0.002
Range	0–7	0–5	
RBS-R Compulsive Score	8.07 (5.43)	2.63 (4.44)	0.005
Range	0–17	0–16	
RBS-R Ritualistic Score	6.13 (5.30)	1.38 (2.63)	0.003
Range	0–16	0–10	
RBS-R Sameness Score	10.93 (7.80)	1.88 (2.90)	<0.001
Range	2–24	0–11	
RBS-R Restricted Score	4.87 (3.00)	0.94 (1.34)	<0.001
Range	0–10	0–5	

Means, standard deviations, and ranges for demographic and characteristic variables by diagnostic group.

Both groups completed the Wechsler Abbreviated Scale of Intelligence (WASI; Wechsler, 1999), as a brief measure of intellectual functioning, the Repetitive Behavior Scale-Revised (RBS-R; Bodfish et al., 1999, 2000) to assess self-reported severity of repetitive behaviors, and the Inventory for Repetitive Behavior (IRB; Bodfish, 2003; Boyd et al., 2013) to evaluate the presence of circumscribed interests. The autistic group completed the Autism Diagnostic Observation Schedule - Generic (ADOS-G; Lord et al., 2000) to confirm criteria for autism spectrum disorder. Additional measures are reported in our previous paper (Richey et al., 2015). Following eligibility, participants completed a cognitive reappraisal training session and a cognitive reappraisal fMRI task with simultaneous eye-tracking and pupil data collection.

### Measures

#### Autism diagnostic observation schedule – G

The ADOS-G is a semi-structured standardized diagnostic measure designed to assess the domains of Communication, Social Interaction, and Stereotyped Behaviors and Restricted Interests (Lord et al., 2000). Trained clinicians observed and coded behaviors, which were loaded onto algorithm scores for each domain. The Stereotyped Behaviors and Restricted Interests score was used for brain-behavior correlations.

#### Wechsler abbreviated scale of intelligence

The WASI (Wechsler, 1999) is a brief clinician-administered assessment for intelligence. The four subscale version of the Full Scale intelligence quotient (FSIQ-4) standard score was used to characterize the sample.

#### Inventory for repetitive behavior

The IRB (Bodfish, 2003) is a brief semi-structured interview used to evaluate the (1) frequency, (2) intensity, (3) interference, (4) accommodation, and (5) peculiarity of repetitive behaviors and interests. Trained interviewers assign a severity score on a Likert scale (0–4) in each of domains listed above on (1) Motor Stereotypies, (2) Insistence on Sameness, and (3) Circumscribed Interests. Total scores range from 0–25, with higher scores indicating more functional impairment. This measure was used to confirm the presence of CI and determine the personalized stimuli to be used in the paradigm. The CI total impairment rating was used for brain-behavior correlations.

#### Repetitive behavior scale-revised

The RBS-R (Bodfish et al., 1999) is a 43-item self-report questionnaire that measures both the presence and severity of repetitive behaviors. Each item is rated on a four-point scale ranging from zero “behavior does not occur” to three “behavior occurs and is a severe problem.” Higher scores indicate more severe repetitive behaviors. The measure was developed with six subscales: Stereotyped, Self-injurious, Compulsive, Ritualistic, Sameness, and Restricted behavior. The RBS-R total score was used for brain-behavior correlations.

### Cognitive reappraisal training sessions

Before undergoing the imaging portion of the study, all participants completed a cognitive reappraisal training session conducted in a one-on-one format with a clinical psychologist (J.A.R.). The cognitive reappraisal training consisted of three phases: First, the experimenter explained the cognitive reappraisal strategies using several sample images not used in the fMRI task. Participants were instructed to reinterpret the meaning of the image in a way that changed their emotional reactions to the picture. To “think positive” about a picture, participants were instructed to imagine the picture is of something they are interested in, something they really like. To “think negative” about an image, they were instructed to imagine that the picture is of something they do not like. Participants were reminded that they may be asked to “think negative” (i.e., downregulate) about pictures that they liked and to “think positive” (i.e., upregulate) about pictures that they did not like. Both self-focused and situation-focused reappraisal strategies were permitted (Ochsner et al., 2004). Participants were also told not to look away from images, not to distract themselves, and not to close their eyes as ways to modify the emotional responses. Second, participants worked collaboratively with the experimenter to practice generating appropriate cognitive reappraisal strategies in the context of several additional images (also not drawn from the fMRI paradigm). During this phase, participants were asked to generate and verbalize a cognitive reappraisal strategy and feedback was provided regarding the appropriateness of each attempt. Examples of correct responses (e.g., describing or interpreting the stimulus in the instructed emotional direction) included the following: For “think positive”: “I would think of a positive experience I had with [object/image].” For “think negative”: “I would think about bad things that could happen with [object/image].” Conversely, examples of incorrect responses (e.g., using emotional terms inconsistent with the instructed direction) included the following: For “think positive”: “It’s useful.” For “think negative”: “I don’t like [object/image].” Finally, to verify cognitive reappraisal comprehension, twelve additional practice images were shown and participants were asked to generate and verbalize examples of cognitive reappraisal strategies independently. Two autistic participants who otherwise met inclusion criteria for the study did not demonstrate adequate comprehension on at least 10/12 practice trials and thus were not scanned, resulting in a final sample of 15 autistic participants who participated in the fMRI portion of the study.

### Cognitive reappraisal functional magnetic resonance imaging task

**FIGURE 1:**
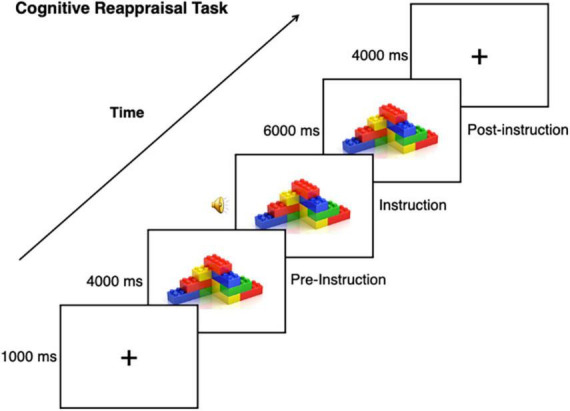
Cognitive reappraisal task. This task was used with CI and nonsocial object stimuli, task instructions included prompting to either “Think Positive” or “Think Negative” about the stimulus.

For details on fMRI acquisition see our previous study (Richey et al., 2015). A modified version of a standard cognitive reappraisal task was used (e.g., Heller et al., 2009), wherein participants viewed a stimulus before and while implementing cognitive reappraisal (Figure 1). For each participant, a total of 10 nonsocial object stimuli were used for the task, which were randomized from a set of 40 nonsocial object images (Sasson et al., 2008). Autistic participants were asked to bring in 10 pictures of their personalized CI, as determined by the IRB, to be used as task stimuli for the autistic participant and matched neurotypical participant. CI categories included: Video/computer games (*n* = 3), Anime (*n* = 1), Music/Band (*n* = 1), Actors (*n* = 2), Vehicles (*n* = 3), Tools/Mechanics (*n* = 4), City (*n* = 1). Stimuli were presented using E-Prime software version 2.0 (Psychology Software Tools Inc., Pittsburgh, PA). Trials began with a 1 s fixation coupled with an orienting tone, after which an image was presented for 10 s. 4 s after image onset, audio prompts to either “Think Positive” or “Think Negative” signaled the participant to engage a specific cognitive reappraisal strategy. Across four runs that were each 480 s in length, 40 images were presented: 16 were presented with instructions to “Think Positive”; 16 were presented with instructions to “Think Negative”; and eight additional trials were presented with instructions to “Look” at the image to reduce predictability. As the “Look” condition was previously found to be underpowered in our previous study, we excluded this condition from our analyses (Richey et al., 2015).

#### Picture ratings

After scanning, participants completed a picture rating task outside of the scanner which inquired about valence and arousal ratings using the self-assessment mannequin (Bradley and Lang, 1994).

#### Eye tracking and pupillometry measures

Eye tracking and pupil responses were simultaneously collected at 60 Hz while participants completed the cognitive reappraisal fMRI task. Details on acquisition and pre-processing of pupillometry and eye tracking are outlined in our previous paper (Richey et al., 2015). Pupillary responses are an index of arousal and of cognitive effort (Cabestrero et al., 2009), which are linked to cognitive reappraisal, such that expanded pupil diameter corresponds to greater expended effort (Johnstone et al., 2007; van Reekum et al., 2007). The mean diameter for the half-second prior to regulation instructions were subtracted from the mean pupil diameter during each half-second picture periods following cognitive reappraisal instructions, and the proportional change in pupil diameter was then computed for each of the time points following the instruction [i.e., (post–pre)/pre]. Thus, negative change in pupil diameter indicates more constriction, whereas positive change in pupil diameter indicates greater dilation. Variables of interest for between group analyses included percentage of time spent on images and average change in pupil diameter response for each condition.

### Data analysis plan

#### Statistical analysis

For Affective Ratings, we conducted two (Valence, Arousal), 2 (Group: Neurotypical, Autistic) x 2 (Stimulus: CI, Nonsocial object) x 3 (Condition: “Pre-Regulation”, “Think Negative”, and “Think Positive”) repeated measures ANOVAs. Follow-up *t*-tests were Bonferroni corrected. We used the same statistical approach for pupillometry and eye tracking data.

#### Functional magnetic resonance imaging analysis

Functional image processing and statistical analyses were then completed using FMRIB’s Software Library (FSL), version 5.0.10 with the FMRIB’s Expert Analysis Tool (FEAT; Smith et al., 2004; Jenkinson et al., 2012). At the first-level, each task condition (i.e., CI Pre-Regulation, CI Enhance Positive, CI Enhance Negative, CI Both Regulation, Nonsocial Pre-Regulation, Nonsocial Enhance Positive, Nonsocial Enhance Negative, Nonsocial Both Regulation) was coded as an explanatory variable (EV) and convolved with a double gamma function, along with its temporal derivative. Each EV yielded a per-voxel parameter estimate (β map) that represented the activation magnitude associated with that regressor. For comparisons of interest, β maps were contrasted. Functional data were registered to MNI stereotactic space using affine transformations. Second-level analyses (i.e., collapsing runs within-subject) used a fixed-effects model. Third-level between-group analyses used FMRIB’s linear analysis of mixed effects (FLAME1 + 2) for each contrast of interest (i.e., CI Regulation vs. Nonsocial Regulation), followed by two-sample *t*-tests (Autistic vs. Neurotypical Group). Due to our small sample size, whole-brain *Z*-statistic maps were cluster defined using a threshold of *Z* ≥ 2.6 (*p* < 0.005) and cluster-corrected using Gaussian random field theory (RFT) at *p* ≤ 0.05.

#### *Post-hoc* generalized psychophysiological interaction analysis

Generalized psychophysiological interaction reveals how brain activity from a particular seed region is differentially correlated with specific brain areas depending on task conditions (i.e., Enhance Positive, Enhance Negative; McLaren et al., 2012). In order to explore the role of the PCC in ER toward CI in autism, a mask of the PCC cluster from the CI Regulation vs. CI Pre-Regulation (MNI_peak_ = 44, 40, 56) contrast was extracted and binerized, and then multiplied by a binarized structural PCC mask from the Harvard-Oxford cortical atlas. Individual PCC time series data for each participant’s runs were extracted using *fslmeants*. FSL FEAT was used to conduct the gPPI analyses. Following similar methods to (Harrison et al., 2017), the first-level FEAT design modeled the original EVs of the task (Richey et al., 2015), PCC time series data, six MCFLIRT motion regressors, and a total of 6 psychophysiological interactions with each condition of interest (i.e., CI and Nonsocial conditions of Pre-Regulation, Enhance Positive, and Enhance Negative). A confound EV was added from the framewise displacement matrix created by *fsl_motion_outliers*. Moreover, following methods from Diano et al. (2017), a GLM contrast comparing each regulation condition’s PPI against the pre-regulation condition’s PPI was computed. These contrasts allowed for the examination of the unique contribution of the regulation condition by discounting the unspecific effects of the pre-regulation condition. Second-level analyses used a fixed-effects model to combine the participant’s four run results together. The final, group-level analysis collapsed participants by group and used FLAME1 + 2. As these *post-hoc* analyses were exploratory, the results were cluster-corrected for multiple comparisons at the whole-brain level of Z >2.3, *p* < 0.01.

#### Brain-behavior correlations

In order to examine brain-behavior relationships within the autistic group, we extracted parameter estimates for six anatomically-derived ROIs of bilateral nucleus accumbens, caudate, and putamen from the Harvard-Oxford subcortical probabilistic atlas (Kötter et al., 2001) from the CI Regulation > CI Pre-Regulation contrast. Additionally, we conducted *post-hoc* exploratory correlations of peak (5 mm) posterior cingulate cortex (PCC) from the CI Regulation > CI Pre-Regulation and CI Pre-Regulation > Nonsocial Pre-regulation contrasts. We used spearman’s rank correlations to examine the link between brain activity with ADOS-G Stereotyped Behaviors and Restricted Interests domain (Sears et al., 1999) and Pearson’s correlations to examine the link between brain activity with RBS-R and IRB scores.

## Results

### Affective ratings

#### Valence ratings

**FIGURE 2:**
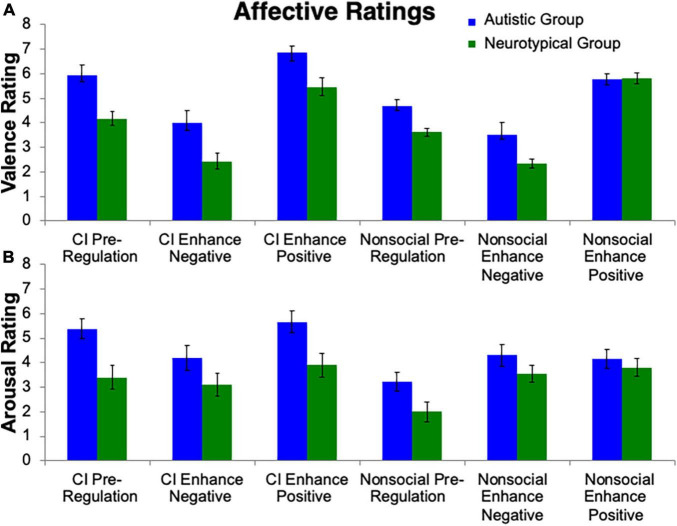
Affective ratings. Valence and arousal ratings toward stimuli were collected. Group differences emerged for **(A)** valence ratings and **(B)** arousal ratings, i.e., that the autistic group reported greater valence and arousal than controls. Additionally, the autistic group reported greater valence and arousal toward CI images than nonsocial object images.

To test the hypothesis that the autistic group, compared to neurotypical group, would demonstrate greater positive affect to the CI than nonsocial object stimuli, a repeated measures ANOVA was conducted (Figure 2). A main effect of Group (*F*(1, 24) = 16.61, *p* < 0.001, η_p_^2^ = 0.41) was observed, with the autistic group reporting greater valence than the neurotypical group. A main effect of Stimulus (*F*(1, 24) = 10.42, *p* = 0.004, η_p_^2^ = 0.31) was found, with the CI images having higher valence ratings than the nonsocial object images. There was also a main effect of Condition (*F*(1, 24) = 61.60, *p* < 0.001, η_p_^2^ = 0.72). Follow-up Bonferroni-corrected *t*-tests revealed significant differences amongst all conditions (all *p*s < 0.001) with the highest valence ratings for Enhance Positive, followed by Pre-Regulation, and then Enhance Negative. Significant interactions were found for Group-by-Stimulus (*F*(1, 24) = 7.07, *p* = 0.02, η_p_^2^ = 0.23), but not for Group-by-Condition (*p* > 0.29). Additionally there was a Stimulus-by-Condition interaction (*F*(1, 24) = 4.24, *p* = 0.02, η_p_^2^ = 0.15) and a trend for Group-by-Stimulus-by-Condition interaction (*F*(1, 24) = 2.71, *p* = 0.08, η_p_^2^ = 0.10).

#### Arousal ratings

The repeated measures ANOVA for arousal ratings (Figure 2) revealed a similar pattern to the valence ratings. A main effect of Group (*F*(1, 24) = 6.05, *p* = 0.02, η_p_^2^ = 0.20), Stimulus (*F*(1, 24) = 19.75, *p* < 0.001, η_p_^2^ = 0.45) and Condition (*F*(1, 24) = 9.12, *p* < 0.001, η_p_^2^ = 0.28) were found. Significant interactions were found for Group-by-Stimulus (*F*(1, 24) = 5.83, *p* = 0.02, η_p_^2^ = 0.20), but not for Group-by-Condition (*p* > 0.24). Additionally there was a Stimulus-by-Condition interaction (*F*(1, 24) = 17.31, *p* < 0.001, η_p_^2^ = 0.15). No interaction was found for Group-by-Stimulus-by-Condition (*p* > 0.31).

### Pupillometry and eye tracking

#### Pupillometry

**FIGURE 3:**
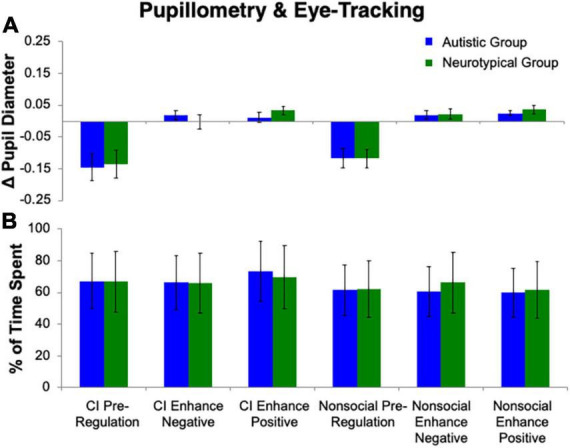
Pupillometry and eye tracking. No group differences emerged for **(A)** change in pupil diameter or **(B)** percentage of time spent on stimuli.

The repeated measures ANOVA for pupillary responses (Figure 3) revealed no effect of Group (*p* > 0.78). A main effect of Stimulus (*F*(1, 25) = 7.05, *p* = 0.01, η_p_^2^ = 0.22) was found, with a greater difference in pupillary diameter for CI than nonsocial object images, such that there was more pupillary constriction for CI than nonsocial images. There was also a main effect of Condition (*F*(2, 24) = 22.01, *p* < 0.001, η_*p*_^2^ = 0.65). Follow-up Bonferroni-corrected *t*-tests revealed significant differences in pupil diameter between Enhance Positive, and Enhance Negative, as compared to Pre-Regulation (*p*s < 0.001). No difference was found in pupil diameter for Enhance Positive vs. Enhance Negative (*p* = 0.75). No significant interactions were found (all *p*s > 0.42).

#### Eye tracking

The repeated measures ANOVA for percent of time spent looking at the image (Figure 3) revealed no effect of Group (*p* > 0.94) or Condition (*p* = 0.50). A main effect of Stimulus (*F*(1, 25) = 8.90, *p* = 0.006, η_p_^2^ = 0.26) was found, with a greater percentage of time spent on CI than nonsocial object images. No significant interactions were found (all *p*s > 0.12).

### Functional magnetic resonance imaging

#### Group differences in omnibus contrasts

In order to test between-group differences in the omnibus contrast we collapsed nonsocial object and CI conditions and examined Enhance Positive > Pre-regulation, Enhance Negative > Pre-Regulation, and Both Regulation > Pre-regulation. Whole-brain analysis revealed that when compared to the neurotypical group, the autistic group demonstrated decreased activation of the L Hippocampus (MNI_peak_ =−32, −40, 0; *Z_*max*_* = 3.39; *k* = 220) when engaging in positive regulation as compared to pre-regulation. Additionally, compared to the neurotypical group, the autistic group demonstrated increased activation in the L Supramarginal Gyrus (MNI_peak_ =−66, −44, 32; *Z_*max*_* = 4.13; *k* = 224) when engaging in negative regulation as compared to pre-regulation. No between-group differences were present when comparing both regulation conditions (enhance negative and enhance positive) to pre-regulation.

#### Group differences in main effects (Enhance positive, enhance negative, pre-regulation, both regulation) by stimulus type

##### Group differences in main effects of nonsocial objects

When engaging in positive regulation of nonsocial object images, between-group whole-brain analysis revealed that as compared to the neurotypical group, the autistic group demonstrated decreased activation in the L Lateral Occipital Cortex (MNI_peak_ =−6, −72, 60; *Z_*max*_* = 4.64; *k* = 225). When engaging in negative regulation with nonsocial object images, the autistic group demonstrated decreased activation in the R IFG (MNI_peak_ = 48, 40, 2; *Z_*max*_* = 3.56; *k* = 302) compared to the neurotypical group. No between-group differences were present for pre-regulation or both regulation conditions of nonsocial object images.

##### Group differences in main effects of circumscribed interests

For pre-regulation of CI images, the autistic group demonstrated greater activation of the PCC (MNI_peak_ = 43, 44, 54; *Z_*max*_* = 3.82; *k* = 471) compared to the neurotypical group. For positive regulation of CI images, the autistic group demonstrated decreased activation in the L MFG (MNI_peak_ = 4, −38, 36; *Z_*max*_* = 3.65; *k* = 188) compared to the neurotypical group. No between-group differences were present for negative regulation of CI images. For both regulation, the autistic group demonstrated less activation in the L MFG (MNI_peak_ =−36, 10, 42; *Z_*max*_* = 3.22; *k* = 414), L SFG (MNI_peak_ =−6, 52, 50; *Z_*max*_* = 3.32; *k* = 269), and L Frontal Pole (MNI_peak_ =−46, 52, −12; *Z_*max*_* = 3.55; *k* = 239) compared to the neurotypical group.

#### Group differences in regulation vs. pre-regulation contrasts by stimulus type

##### Group differences in regulation vs. pre-regulation of nonsocial objects

Lower order interactions were tested, which were organized by stimulus type to assess between-group differences in whole-brain activation. There were no between-group differences when comparing positive regulation, negative regulation, and both regulation to pre-regulation of nonsocial object images.

##### Group differences in regulation vs. pre-regulation of circumscribed interests

**FIGURE 4:**
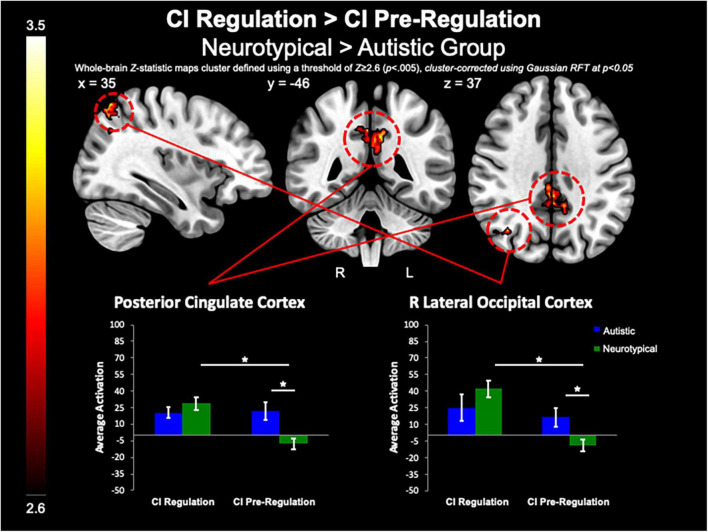
CI regulation > CI pre-regulation. Compared to controls, the autistic group demonstrated decreased activation in the PCC and R lateral occipital cortex when regulating CI images vs. viewing CI images.

The between-group whole-brain analysis revealed that when compared to the neurotypical group, the autistic group demonstrated decreased activation in the PCC (MNI_peak_ = 2, −46, 40; *Z_*max*_* = 3.52; *k* = 507) and R lateral occipital cortex (MNI_peak_ = 36, −64, 62; *Z_*max*_* = 3.72; *k* = 286) for both regulation as compared to pre-regulation of CI images (Figure 4). Further, the PCC (MNI_peak_ = 2, −44, 34; *Z_*max*_* = 3.72; *k* = 265) was less active in the autistic group vs. neurotypical group for positive regulation as compared to pre-regulation of CI images. Similarly, compared to the neurotypical group, the autistic group demonstrated less activation of the PCC (MNI_peak_ =−14, −64, 30; *Z_*max*_* = 3.67; *k* = 728) for negative regulation as compared to pre-regulation of CI. Further examination of these differences shows that group differences in these contrasts were related to lack of modulation of these areas in the autistic group, while the neurotypical group demonstrated greater activation of these areas during regulation, and decreased activation of these areas during pre-regulation (Figure 4).

#### Group differences in circumscribed interests vs. nonsocial object contrasts

**FIGURE 5:**
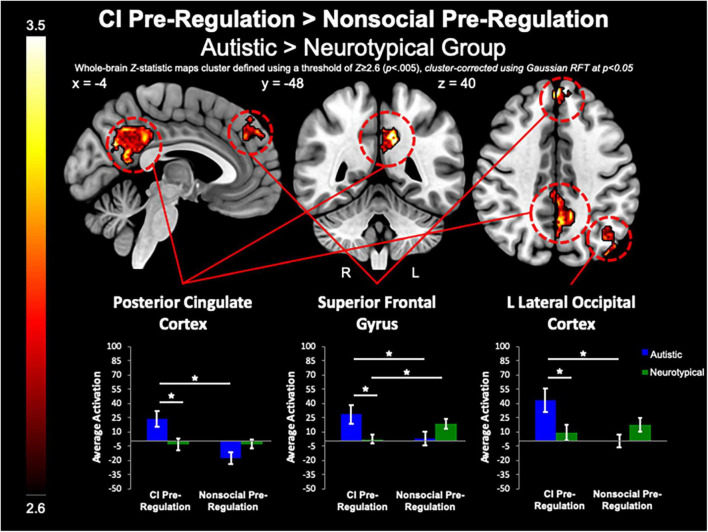
CI pre-regulation > nonsocial pre-regulation. Compared to controls, the autistic group demonstrated increased activation in the PCC and SFG, and L Lateral Occipital Cortex when viewing CI images vs. viewing nonsocial object images.

**FIGURE 6:**
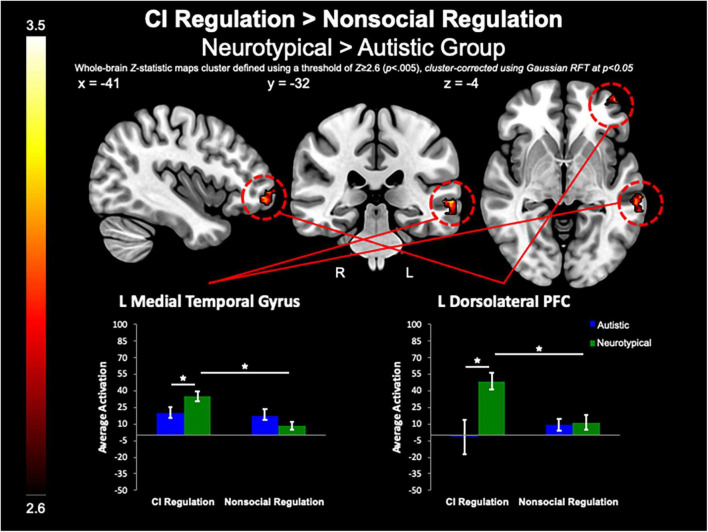
CI regulation > nonsocial regulation. Compared to the autistic group, the control group demonstrated increased activation of the L MTG, and dlPFC when regulating affect toward CI vs. regulating affect toward nonsocial object images.

Between-group whole-brain analysis revealed that compared to the neurotypical group, the autistic group demonstrated greater activation of the PCC (MNI_peak_ =−12, −48, 32; *Z_*max*_* = 3.78; *k* = 818), L lateral occipital cortex (MNI_peak_ =−44, −74, 46; *Z_*max*_* = 3.98; *k* = 365), and SFG (MNI_peak_ = 0, 48, 40; *Z_*max*_* = 3.64; *k* = 224) during the pre-regulation period for CI images as compared to the pre-regulation period for nonsocial object images (Figure 5). The neurotypical group evidenced greater activation than the autistic group in the L frontal pole (MNI_peak_ =−50, 54, −8; *Z_*max*_* = 3.45; *k* = 256) and L MTG (MNI_peak_ =−60, −30, −2; *Z_*max*_* = 3.4; *k* = 236) when regulating CI as compared to vs. regulating nonsocial object images (Figure 6). There were no other between-group differences when comparing CI vs. nonsocial objects.

### Group differences in functional connectivity

A gPPI analysis was conducted to examine a functionally-defined PCC connectivity to the whole-brain during both regulation (enhance positive and enhance negative) compared to pre-regulation of CI images. As compared to the neurotypical group, the autistic group demonstrated greater coupling of the PCC with the L lateral occipital cortex (MNI_peak_ =−22, −78, 40; *Z_*max*_* = 3.51; *k* = 1218) and R Supramarginal Gyrus (MNI_peak_ = 54, −32, 52; *Z_*max*_* = 4.05; *k* = 620), during both regulation conditions as compared to pre-regulation of CI images.

### Correlations between brain activation and repetitive behaviors

**FIGURE 7:**
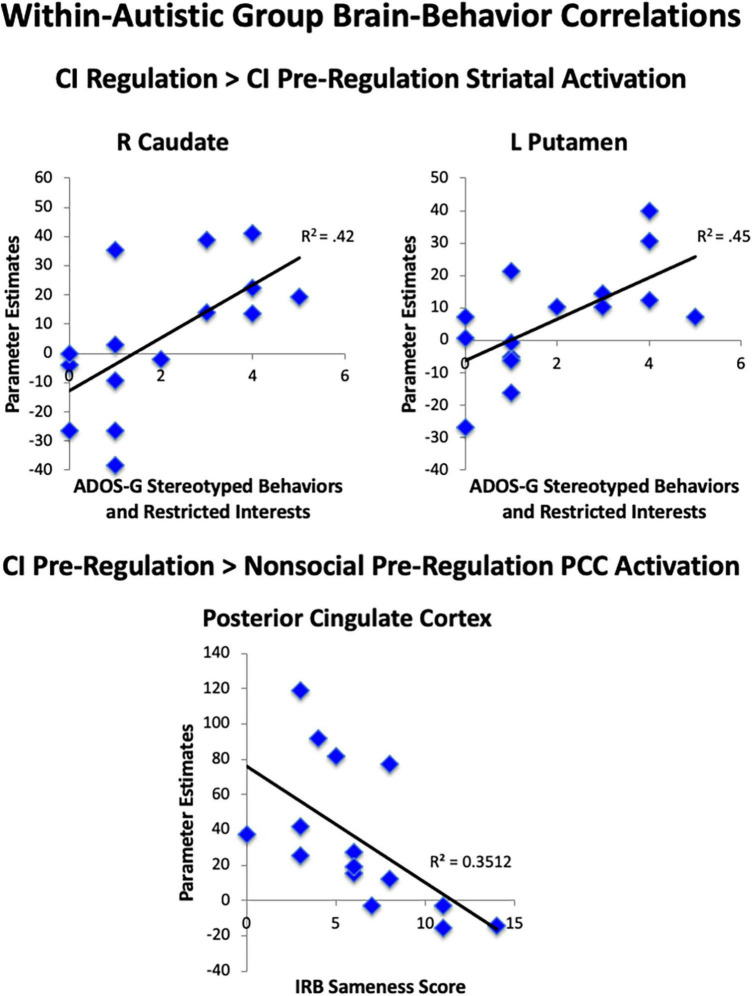
Brain-behavior correlations. Within the autistic group, correlations were found between less striatal area activation during viewing of CI images and greater RRBI. Correlations were found for greater striatal area activation when regulating CI than when viewing CI and greater RRBI.

We examined parameter estimates, which were averaged across anatomically-defined ROIs of the nucleus accumbens, caudate, and putamen to test our *a priori* hypotheses that striatal activation during the task would be linked to RRBI in the autistic group. When comparing both regulation conditions to pre-regulation of CI, we found significant correlations between the Stereotyped Behaviors and Restricted Interests domain of the ADOS-G and R caudate (rho = 0.65, *p* = 0.009), and L putamen (rho = 0.67, *p* = 0.007; Figure 7), with higher repetitive behaviors associated with greater increase in striatal activation while asked to regulate response to CI than when viewing CI. Exploratory *post-hoc* correlations of peak PCC activation revealed that greater decrease in PCC activation for CI pre-regulation as compared to nonsocial object pre-regulation was correlated with IRB sameness scores (*r* =−0.59, *p* = 0.02), such that the greater the insistence on sameness, the less PCC activation was observed when viewing CI images relative to nonsocial objects.

## Discussion

This study examined biological and behavioral mechanisms of emotion regulation toward highly motivating contexts (i.e., CI and nonsocial object images) in autistic adults. In the present study, different levels of behavioral and biometric data revealed differences in emotion regulation toward CI and nonsocial objects. On a behavioral level, autistic participants reported greater overall positive affect and greater arousal to CI stimuli compared to neurotypical participants. Typically, greater arousal and interest is associated with increased pupil size and looking time. However, no group differences in eye tracking or pupillometry data were observed in the present study. On a neural level, group differences were observed both when collapsing CI and nonsocial objects, as well as in response to viewing CI without regulation instructions. Finally, neural patterns of activation were associated with measures of RRBI. Each level of analysis is informative for understanding the mechanisms of emotion regulation specific to highly motivating stimuli in autistic adults.

In the present study, autistic participants demonstrated greater activation in the PCC compared to neurotypical participants across multiple contexts related to viewing their CI (i.e., pre-regulation), including group differences in comparison to viewing nonsocial objects, and regulation conditions. Notably, the PCC is not known as part of the neural response to rewarding stimuli, and therefore it was not a hypothesized region of interest. The use of the PCC to process CI is significant, as the PCC is increasingly associated with resting-state brain networks, and personalized processing. Recruitment of the PCC may specifically relate to the stimuli we used and its interaction with systems that modulate ER. First, the PCC has been demonstrated to be involved in attention to self- (i.e., autobiographical memory, imagining future self) and other-referential processing (i.e., evaluating and processing other’s mental states; Brewer et al., 2013). As personalized CI are highly intrinsically motivating (Grove et al., 2018) and qualitative work has noted that CI in autism are intertwined with self-images (Winter-Messiers, 2007), the self-referential role of CI may be modulated differently in the PCC for autistic people compared to neurotypical people. Second, the PCC is a main hub of the default mode network (DMN), which is a brain network that *decreases* in activity when one is focused on a task, and *increases* in activity when engaging in “internally focused tasks,” i.e., letting one’s mind wander (Buckner et al., 2008; Greicius et al., 2009). Our findings of increased activation during pre-regulation of CI, and lack of modulation of the PCC when regulating, suggest that CI may elicit ER responses akin to the absence of task demands and similar to relaxed state for autistic people. Moreover, although PCC recruitment for cognitive reappraisal is not commonly found, with most work supporting frontolimbic areas (Buhle et al., 2014), previous cross sectional work by McRae et al. (2012) found that the PCC was more active during regulation for adolescents ages 14–17 years, as compared to both children and adults. There is a need in parsing the role of the PCC in autism as it relates to ER mechanisms, as several studies have found alterations with the PCC and DMN in autistic samples, with alterations frequently linked to autistic traits (Spencer et al., 2012; Lynch et al., 2013; Yerys et al., 2015; Padmanabhan et al., 2017). Further, recent work has emphasized the role of the DMN and PCC in ER difficulties (i.e., repetitive negative thinking, internalizing symptomology) for autistic people (Burrows et al., 2017; Hogeveen et al., 2018). Thus, alterations in PCC activation toward CI stimuli add to our understanding of altered ER mechanisms for autistic people.

Although there are many positive aspects to CI for autistic people, including a sense of satisfaction and wellbeing, very high intensity of CI has been linked with poorer subjective wellbeing in autistic adults, such that those with very high levels of time spent on CI reported overall less satisfaction, less happiness, and poorer quality of life than those that spent less time on CI (Grove et al., 2018). Moreover, higher interest intensity has been linked to greater social, adaptive, and executive function difficulties (Anthony et al., 2013). Grove et al. (2018) suggested two potential reasons that very high intensity of CI and negative outcomes may be related: (a) there may be a “trade-off” with very high intensity CI impacting negatively on well-being, or (b) there may be greater use of CI as a means to cope with high levels of distress. The latter is consistent with recent qualitative work suggesting that CI may be an emotion regulation strategy to prevent autistic burnout, or chronic exhaustion, decreased ability to function, and increased sensory sensitivity due to masking of autistic traits or life stressors (Raymaker et al., 2020; Higgins et al., 2021; Mantzalas et al., 2022a,b). More work is needed in examining these hypotheses, and it may be the case that both patterns exist for different people, at different levels, or at different times.

Our brain correlations support a link between intensity of RRBI and emotion regulation mechanisms, such that higher clinician-reported RRBI was linked to greater reward activation during cognitive reappraisal vs. pre-regulation of CI in the autistic group, indicating a potential difficulty in regulating reward areas when engaging in ER strategies. Moreover, decreased PCC activation when comparing viewing CI to viewing nonsocial images was linked to greater insistence on sameness, suggesting an association between modulation of the PCC toward various types of information and insistence on sameness for autistic people. This finding is consistent with work linking repetitive thinking to neural mechanisms of internalizing symptomology in autism (Burrows et al., 2017; Hogeveen et al., 2018). Altogether, these results support that neural mechanisms of ER toward CI may be uniquely linked to RRBI.

These findings are largely consistent with prevailing ER frameworks suggesting that recruitment of cognitive control areas including dlPFC are related to experienced emotion (Ochsner and Gross, 2005). A meta-analysis by Buhle et al. (2014) found that dorsolateral PFC, dorsomedial PFC, ventrolateral PFC, and posterior parietal areas were active during cognitive reappraisal of both positively-valenced and negatively-valenced stimuli, and comparatively, that greater amygdala activation was present during natural viewing. Atypical ER mechanisms have been found in autsitic vs. neurotypical people when regulating emotionally evocative stimuli (Pitskel et al., 2014) and neutral face stimuli (Richey et al., 2015) with cognitive reappraisal tasks. Specifically, alterations in prefrontal and limbic areas have been supported. This study converges with previous work in that the autistic group demonstrated decreased activation of PFC areas for regulation of both CI and nonsocial object images compared to the neurotypical group. Additionally, although the two previous studies have found decreased amygdala activation during cognitive reappraisal in autism vs. neurotypicals (Pitskel et al., 2014; Richey et al., 2015), we did not find this effect. Lack of differences in amygdala activation may be related to stimuli used in this study. Previous studies have used stimuli which are sensitive to threat and social salience, which may explain magnify the role of amygdala reactivity during ER (Phelps and LeDoux, 2005; Schultz, 2005; Herrington et al., 2015).

More work is needed in understanding how positively-valenced stimuli may impact ER systems (Buhle et al., 2014). In autism, there is limited work on emotion regulation of highly positive stimuli, with work focusing on positive social information. For example, one study highlighted that autistic and neurotypical persons report similar affective empathy in response to socially positive stimuli, though group differences are amplified for cognitive empathy, especially when interpreting socially negative stimuli (Mazza et al., 2014). In our current study, greater activation in frontal brain regions of the neurotypical vs. autistic group was found regulation of CI vs. regulation of nonsocial images. Our finding of increased left MFG, SFG, and frontal pole activation in the neurotypical vs. autistic group is consistent with previous results of increased left frontal brain regions when regulating affect toward positive images (i.e., images that elicit joy; Mak et al., 2009). One potential explanation for this difference is that the autistic group did not need the same level of processing to regulate their CI as the neurotypical participants, as they may already have a well-developed system for regulating emotion toward CI.

This study is not without limitations, which are important to note. First, it is a small sample. Due to this small sample size, we used a relatively liberal cluster defining threshold (*p* < 0.005) and future work with larger sample sizes should use conservative cluster defining thresholds or non-parametric permutation testing. Our sample was predominantly comprised of male participants without intellectual disability. It should be noted that within our sample we did not collect data on age of autism diagnosis, current interventions, socioeconomic status, and education level, and this information may have been useful in better characterizing our sample. Moreover, this study specifically screened for the presence of CI and not all autistic people endorse CI (Klin et al., 2007; Grove et al., 2018). Thus, as with any study, the generalizability of these results should be interpreted with caution. Second, we did not have a measure of ER to confirm whether brain activation during cognitive reappraisal related to real-world ER or psychopathology. Though our arousal and valence ratings support changes in affective modulation, it will be important for future work to include real-world ER measures in addition to affective ratings. Third, the study was designed so that all autistic participants viewed their personalized CI, while matched neurotypical participants viewed an autistic participant’s CI. Therefore results related to PCC activation from the CI condition may be conflated with familiarity of personalized CI images within the autistic group. It will be important for future work to include familiar interest images for neurotypicals to better parse the role of the PCC in ER. As CI is a common feature of autism, intensity of interests may not have been possible to match in the general population. Despite this potential intensity difference, previous work has used personalized interest stimuli for neurotypical participants with success and including this type of control in future work will allow for more specific interpretations (e.g., Kohls et al., 2018). Additionally, the present study also included nonsocial object images to attempt to minimize confounds between CI with social interests, as CI are not always nonsocial.

It is important for future work to prioritize understanding how altered ER mechanisms toward social and nonsocial images relate to real-world ER strategies and difficulties (i.e., anxiety, depression), as well as to autistic traits across both social and RRBI domains. Further characterizing these relationships may reveal pathways for risk and resilience as well as targeted intervention. Moreover, considering the altered profiles of RRBI in autistic girls (Antezana et al., 2019) and the elevated emotion regulation difficulties noted in females (Gotham et al., 2014; Hiller et al., 2014; White et al., 2017; Wieckowski et al., 2020), it may be important to understand mechanisms of ER toward CI for autistic females, and its relationship to co-occurring symptomology.

The present study is significant in a number of ways. First, this study characterizes ER mechanisms in autistic adults, whereas much of the current literature to date has examined ER in primarily autistic youth (Cai et al., 2018). Further, this study impresses the importance of measuring multiple units of analysis in order to detect regulation effects. For example, the present eye tracking findings indicate that *both* groups found the CI more interesting and arousing than general nonsocial stimuli, whereas behaviorally the autistic group reported greater positive affect and arousal while viewing the images compared to neurotypical group. Thus, gaining self-reported valence and arousal in autism remains important. Neural responses gave an additional layer of information on the specific mechanisms. Recruitment of the PCC in autistic group while regulating responses to CI has the potential to inform clinical interventions for ER for autism more broadly. Modulation of the PCC has been implicated in several mindfulness-based interventions (Brewer and Garrison, 2014; Kral et al., 2019; Cernasov et al., 2021). Emerging work on mindfulness-based interventions that target ER difficulties in autism have demonstrated promising results (Singh et al., 2011; de Bruin et al., 2015; Conner et al., 2019; Beck et al., 2022; Shaffer et al., 2022) and understanding ER mechanisms which promote outcomes will aid in the specificity of targeted treatments. As our work highlights that the PCC may play an important role in ER mechanisms for autistic people, it will be important for future work to bridge this gap.

## Conclusion

The purpose of this study was to examine multiple levels of ER (i.e., neural activity, eye-tracking/pupillometry, affective ratings) to investigate the mechanisms of ER during passive viewing and cognitive reappraisal of personalized (self-selected) CI and nonsocial object images. Although results did not indicate group differences in eye-tracking/pupillometry, the autistic group demonstrated comparatively reduced modulation of the PCC activation between passive viewing vs. cognitive reappraisal of CI images. Further, the PCC was more active for the autistic vs. neurotypical group when viewing their CI vs. nonsocial object images, while the neurotypical group demonstrated greater frontal activation during regulation of CI vs. nonsocial object images. Moreover, greater PCC coupling with left lateral occipital and right supramarginal areas was found in the autistic vs. neurotypical group. We conclude that in autistic adults, CI may be differentially modulated *via* PCC during ER. In light of the documented role of the PCC as a core hub of the DMN, we further postulate that ER of CI may be linked to enhanced self-referential cognition.

## Data availability statement

The data analyzed in this study is subject to the following licenses/restrictions: Participants did not consent to publicly available data. Requests to access these datasets should be directed to richey@vt.edu.

## Ethics statement

This study involved human participants and was reviewed and approved by the Local Human Investigations Committees at both UNC-Chapel Hill and Duke University Medical Centers. The participants provided written and verbal consent to participate in this study.

## Author contributions

LA led the conception of the study aims, analyses, and initial draft, under the mentorship of JR. LA, MC, AD, and JR were involved in drafting and revisions of the manuscript. All authors contributed to the article and approved the submitted version.
